# Design and Development of a Robotized System Coupled to µCT Imaging for Intratumoral Drug Evaluation in a HCC Mouse Model

**DOI:** 10.1371/journal.pone.0106675

**Published:** 2014-09-09

**Authors:** Gaétan Bour, Fernand Martel, Laurent Goffin, Bernard Bayle, Jacques Gangloff, Marc Aprahamian, Jacques Marescaux, Jean-Marc Egly

**Affiliations:** 1 Institut de Recherche contre les Cancers de l′Appareil Digestif (IRCAD), Strasbourg, France; 2 IGBMC, Department of Functional Genomics and Cancer, CNRS/INSERM/Université de Strasbourg, BP 163, Illkirch, C. U. Strasbourg, Strasbourg, France; 3 ICube laboratory UMR, CNRS 7357, University of Strasbourg, Strasbourg, France; The Ohio State University, United States of America

## Abstract

Hepatocellular carcinoma (HCC) is one of the most common cancer related deaths worldwide. One of the main challenges in cancer treatment is drug delivery to target cancer cells specifically. Preclinical evaluation of intratumoral drugs in orthotopic liver cancer mouse models is difficult, as percutaneous injection hardly can be precisely performed manually. In the present study we have characterized a hepatoma model developing a single tumor nodule by implantation of Hep55.1C cells in the liver of syngeneic C57BL/6J mice. Tumor evolution was followed up by µCT imaging, and at the histological and molecular levels. This orthotopic, poorly differentiated mouse HCC model expressing fibrosis, inflammation and cancer markers was used to assess the efficacy of drugs. We took advantage of the high precision of a previously developed robotized system for automated, image-guided intratumoral needle insertion, to administer every week in the tumor of the Hep55.1C mouse model. A significant tumor growth inhibition was observed using our robotized system, whereas manual intraperitoneal administration had no effect, by comparison to untreated control mice.

## Introduction

Hepatocellular carcinoma (HCC) represents 80–85% of the primary malignant liver tumors and is the 3^rd^ most common cancer related death worldwide, with a high increase in developed countries [1]. One of the main challenges in cancer treatment is the targeting of the drug to the tumor to increase the local concentration of the therapeutic agent and avoid major side effects on healthy tissues [2]. This is addressed by using either specifically formulated drug vehicles [2] or targeted therapies delivered by intravenous administration. Some cancers like early HCC can also be treated by radiofrequency, thermal ablation or percutaneous ethanol or acetic acid injection in the tumor [3]. Moreover, among the recent developments in cancer treatment, viral based gene therapy often requires an intratumoral administration [4]–[7]. Intratumoral injection is also frequently performed in the preclinical steps on animal models to develop and assess the efficacy of new therapies [8]–[13].

For many decades, carcinogenesis studies as well as assays in drug therapy utilized tumor rodent models. The recent development of medical imaging especially micro CT scanner (µCT), allows to follow the evolution of the disease and/or the therapy at high resolution, on the same model animal; it also make it possible to perform preclinical drug evaluation in a non-invasive manner on orthotopic rodent cancer models [14], [15]. Additionally such experimental procedures might avoid the intense use of animal models.

Several types of cancer animal models are reported to study hepatocellular carcinoma [16]: (i) genetically engineered mice developing spontaneous tumors; (ii) chemical induction using carcinogens; (iii) xenograft models, in which HCC cells or tumors from human origin are injected subcutaneously or orthotopically into the liver of immunocompromised mice; (iv) syngeneic graft of rodent HCC cells or tumors; (iv) viral hepatocarcinogenesis. Graft of HCC cell lines is the fastest model as it allows tumor growth within days after implantation [14], [15].

In the present study, we characterized a hepatoma model in immunocompetent mice, obtained by implantation of Hep55.1C cells [17] in the liver of syngeneic C57BL/6J mice. We demonstrated that this mouse model can be used to assess the efficacy of drugs taking advantage of a robotized system developed for automated, image-guided intratumoral administration [18].

## Results

### Development of a restrainer bed system for *in vivo* imaging

**Figure 1:**
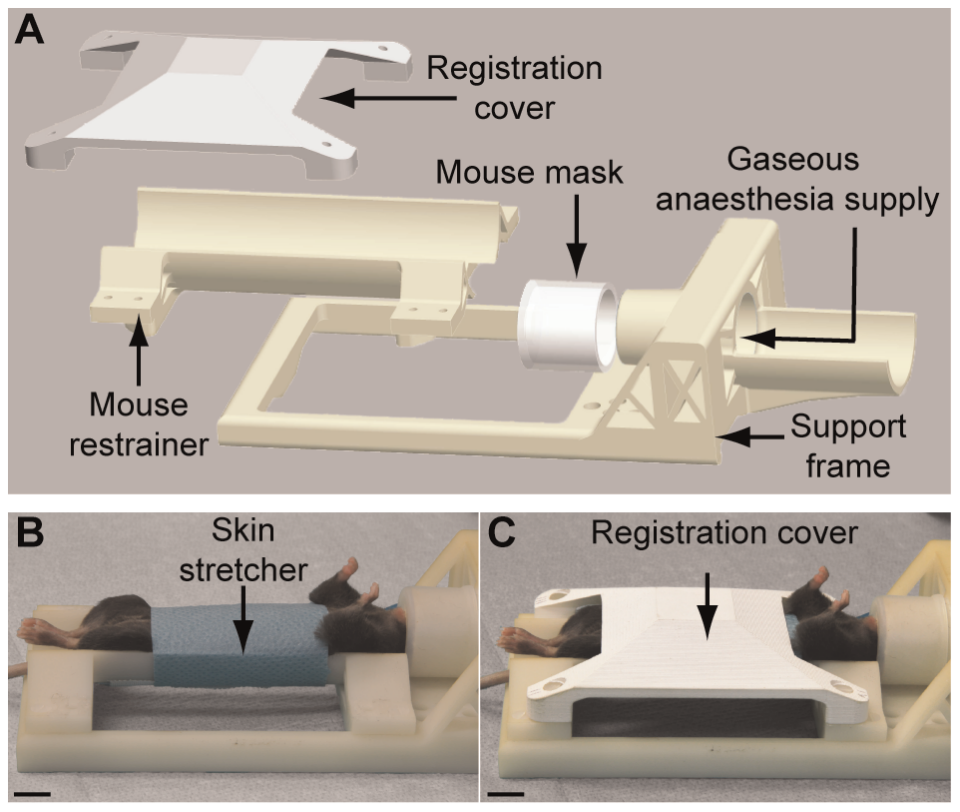
Mouse restrainer and robotized needle injector for intratumoral drug administration. (**A**) Description of the mouse restrainer bed. (**B**) View of a mouse restrained under gaseous anesthesia. The animal is tightly fixed on a hemicylindrical shell by a sterile operative field stuck on the abdomen. A sensor placed on the back of the mouse monitors respiration. (**C**) A dedicated registration cover is screwed on the bed frame during µCT scan imaging and registration by structured light projection.

Since most µCT scanners do not allow to perform robotized procedures under CT guidance in real-time, we designed a robotized-cell and a process to use pre-operative scan data to precisely target the affected organ. However, the use of µCT scanner analysis for further robotized investigations has to be performed in well-defined conditions. First of all, the animal has to be maintained anesthetized during the whole procedure. We then modeled a dedicated bed for 4–8 week-old mice by Computer Assisted Design (Figure 1A). This bed is composed of three different parts: (i) the support frame of the bed that can be placed inside the µCT scanner, lipped on the existing carbon bed for imaging, as well as in the robotized cell for the image-guided robotized intratumoral administration of the drug. The mouse is maintained under gaseous anesthesia during the entire procedure; (ii) the hemi-cylindrical mouse restrainer that is plugged and screwed in the support frame, firmly restraining the animal in a comfortable position on its back (Figure 1B) to expose the abdomen of the mouse. A sterile operative field stuck on the shaved abdomen ensures that the mouse does not slide during the transfer from the scanner to the robotized cell. In addition, tensing the skin and compressing the abdomen facilitates the penetration of the needle during the robotized insertion step; (iii) the removable registration cover [18], [19] screwed on the support frame, overlaps the mouse on the restrainer part (Figure 1C) during the imaging recording after which it will be removed prior to the needle insertion. A needle insertion system was developed [18], keeping into account the size of the animal, the skin elasticity and the internal organ displacement. A particular attention was paid to the choice of the needle, which length, diameter, and bevel must be compatible with either drug injection or biopsy. Except for the final injection, all the steps are automated, from the positioning outside the animal, to the insertion in the animal.

### Hepp55.1C cells induce fast growing orthotopic HCC

**Figure 2:**
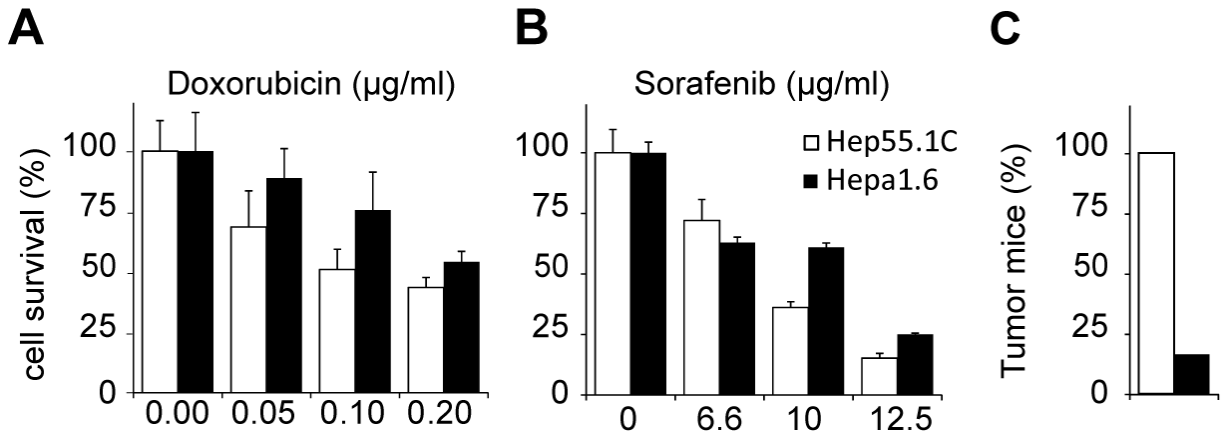
Screening of mouse hepatoma cell lines for chemical sensitivity and tumorigenicity. Hepa1.6 and Hep55.1C cells were exposed to Doxorubicin (**A**) and Sorafenib (**B**) for 3 days and assessed for toxicity using a cell proliferation assay. Results are shown as the mean +/− SD of triplicates. (**C**) Percentage of C57BL6J mice (n = 6) with palpable tumor two weeks after subcutaneous injection of either 10^6^ Hepa1.6 or Hep55.1C in the back mice.

In parallel, to develop a HCC tumor model that would be accepted by immune competent mice, Hep55.1C and Hepa1.6 rodent HCC cell lines [17], [20] were screened for their sensitivity to human standard therapeutic drugs for HCC and their tumorigenicity. We observed that Hep55.1C cells were more sensitive to Doxorubicin and Sorafenib than Hepa1.6 cells: IC50 values for Doxorubicin being around 0.10 µg/ml for Hep55.1C and 0.20 µg/ml for Hepa1.6, and in the range 6.6–10 µg/ml and 10–12.5 µg/ml respectively for Sorafenib (Figure 2A and 2B). Interestingly, Hep55.1C cells were found to be much more tumorigenic than Hepa1.6 cells, as all mice injected subcutaneously with Hep55.1C cells developed tumors, whereas Hepa1.6 tumors were observed in only 15% of the mice (Figure 2C).

**Figure 3:**
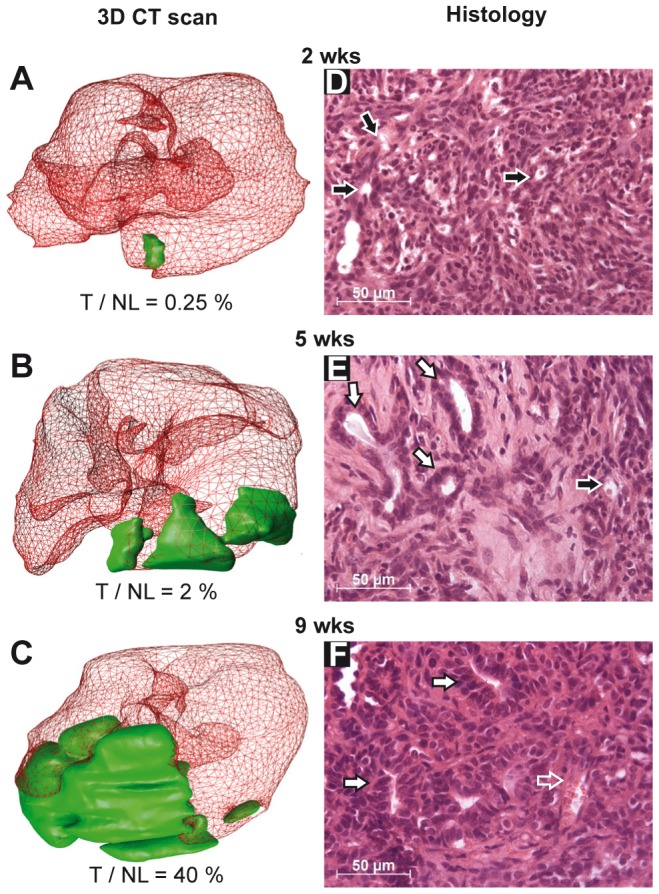
Evolution of tumor after intra liver injection of Hep55.1C cells. Evolution of liver tumor was followed by µCT scan imaging and histology two (**A, D**), five (**B, E**) and nine (**C, F**) weeks after Hep55.1C cells grafted in the liver of C57BL6/J mice. (**A–C**) 3D reconstructions from representative µCT scan showing the normal liver (red) and the tumor (green). Tumor/normal liver volume ratio (T/NL) computed from 3D reconstruction is indicated. (**D–F**) H&E staining of formalin fixed paraffin embedded tumors (black arrow: vesicle, closed white arrow: glandular structure; open white arrow: red blood cells).

We next followed HCC evolution in the Hep55.1C syngeneic orthotopic graft mouse model by recurrent µCT imaging and histological evaluation (Figure 3). An intrahepatic HCC was initiated by injection of 2×10^6^ Hep55.1C cells either directly into the liver left lateral lobe or indirectly via the spleen. Macroscopically, Hep55.1C tumors appeared either as a white large nodule or as multiple small white nodules under liver capsule (). By micro CT imaging, contrast enhancement of the liver allowed a clear identification of the tumor nodules (TN) that remained hypo dense by comparison to normal contrast-enhanced liver (Figure S1C and S1D).

We focused on the intrahepatic graft model that developed single, well localized tumor nodules identified by µCT imaging, and more adapted to evaluate drug. The small nodule (initiated by the Hep55.1C cells) visualized at 2 weeks (in green on Figure 3A), expended progressively in the lobe (Figure 3B). The 3D reconstructions highlight the rapid increase in tumor volume from 5 to 9 weeks, rising from 2% to 40% (Figure 3B–C). Indeed, at 9 weeks, a large nodule invaded the left lateral liver lobe as well as the peritoneal cavity. We observed that the median survival was around 8 weeks, concurrently with the strong increase of tumor size (Figure S2). Similar results were also obtained by grafting Hep55.1C cells in male mice [21].

The progression of the tumor was also followed by histological examination. At 2 weeks after graft, Hep55.1C tumors presented small sized cells with an increasing nuclear/cytoplasm ratio and empty vesicles (right panel black arrows in Figure 3D) that could correspond to vesicular steatosis, in line with positive oil red O staining of tumor frozen section (Figure S3A). At that stage, Hep55.1C tumor cells were highly proliferative, as depicted by a Ki67 index of 40 (fraction of green positive nuclei in figure S3B), comparable to the Ki67 score observed in human HCC [22]. At 5 weeks the tumor exhibited a pseudo glandular aspect similar to a cholangiocarcinoma (Figure 3E, closed white arrows) and presented some features of non-alcoholic steatohepatitis (NASH) with vesicular steatosis (black arrow). At 9 weeks after graft, Hep55.1C tumors corresponded to a poorly differentiated human HCC (Grade III/IV) with a very dense fibrotic structure, and tumor cells with an almost invisible cytoplasm. Some glandular structures and vessels (indicated respectively by open and closed white arrows, Figure 3F) were observed at the periphery of the tumors. The presence of tumor neo-angiogenesis was assessed by immunofluorescence microscopy; we observed a labeling of vessels by the specific endothelial cell marker PECAM-1/CD31 (green staining in figure S3C). In addition, a very strong fibrosis appeared in early and advanced Hep55-1C tumors (Figure 3E–F) as visualized by silver and Sirius red staining for reticulin and collagen respectively (Figure S3D and data not shown). Fibers of collagen and reticulin were scattered throughout the tumor, invading the anarchic Tumor cells and surrounding the pseudo glandular structures (white arrow in figure S3D. The presence of reticulin and collagen fibers inside the tumor is independent of animal sex, fibrosis deposition being observed following orthotopic graft of Hep55.1C cells in both male and female mice (data not shown).

**Figure 4:**
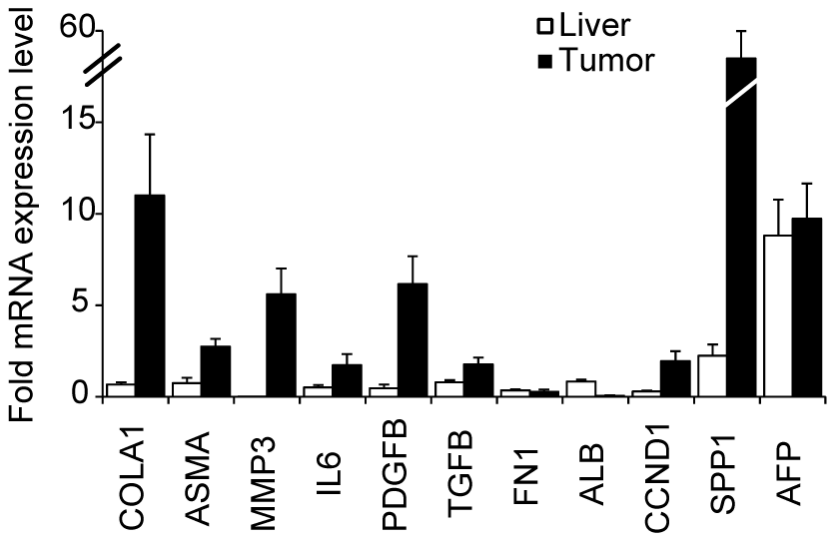
Hep55.1C tumors markers. Expression of COLA1, ASMA, MMP3, FN1 fibrosis markers, IL6, PDGFB, TGFB inflammation markers and ALB, CCND1, SPP1, AFP cancer markers was assessed by RTqPCR from Hep55.1C tumor and corresponding surrounding normal liver. Relative mRNA level of each gene was normalized to the level of the housekeeping gene 36B4. Results are expressed as the mean +/− SEM for at least 3 animals.

We further investigated for the presence of fibrotic genes by RT-qPCR on Hep55.1C tumor extract (Figure 4). We observed a strong expression of type 1 collagen (COLA1), a major extracellular matrix component by comparison to surrounding normal liver cell extract. As collagen is mainly deposited by during fibrosis, the overexpression of both alpha smooth muscle actin (ASMA), and matrix metalloproteinase 3 (MMP3) genes, two activated stellate cells (HSC) markers was noted in tumor cells extract compared to wild type cells. Moreover, an increase in the mRNA level of signaling molecules associated with inflammation and hepatic stellate cell activation like interleukin-6 (IL-6), platelet derived growth factor beta (PDGFB); transforming growth factor beta (TGFB) was also marked by comparison to the surrounding liver cells (Figure 4). However, no expression of fibronectin 1 (FN-1), another extracellular matrix protein and fibrosis marker, was observed. Interestingly, we found that Hep55.1C tumor do not express albumin, a liver specific gene, in line with its poorly differentiated state. On the contrary, cyclin D1 (CCND1), implicated in cell cycle regulation and often dysregulated in cancers, and osteopontin (SPP1) a HCC marker [23]–[25], were highly expressed. The HCC specific marker alpha fetoprotein (AFP) was also overexpressed in the tumor extract (Figure 4), but also in the adjacent liver presumably as a consequence of liver injury and regeneration consecutive to the graft and tumor growth [26].

The graft of Hep55.1C cells in the syngeneic mice liver allows the fast growth of a highly differentiated tumor, expressing fibrotic and cancer marker genes. Pretreatment of the mice with diethylnitrosamine for 4 weeks to induce liver fibrosis prior to Hep55.1C cell graft did not modify tumor growth and marker gene expression (Figure S4 and data not shown).

### Robotized intratumoral injection inhibits Hep55.1C tumor growth

Having set up and followed the evolution of HCC tumor in mice, we evaluated the potency of intratumoral drug administration, using our system for image-guided robotized needle positioning [18]. µCT scan imaging helps to precisely position the needle of a disposable syringe filed with a test drug at the center of the tumor; the needle is positioned by a robot with a deviation lower than 0.3 mm (Figure S5 and [18]). We compared the growth of hepatic tumors in untreated mice to mice either treated intratumorally (IT) with Doxorubicin using our automated positioning system, or injected intraperitoneally (IP). For intratumoral injection, a 29G needle was used to facilitate the insertion while limiting damaging effects on tumors. In addition, it has been demonstrated in previous studies that the sole intratumoral injection of inactive compounds in rodent models of human cancers did not inhibit tumor growth [27], [28]. Thus we postulated that a reduced tumor growth in the IT group could be attributed to the antineoplastic effect of Doxorubicin on tumor cells.

**Figure 5:**
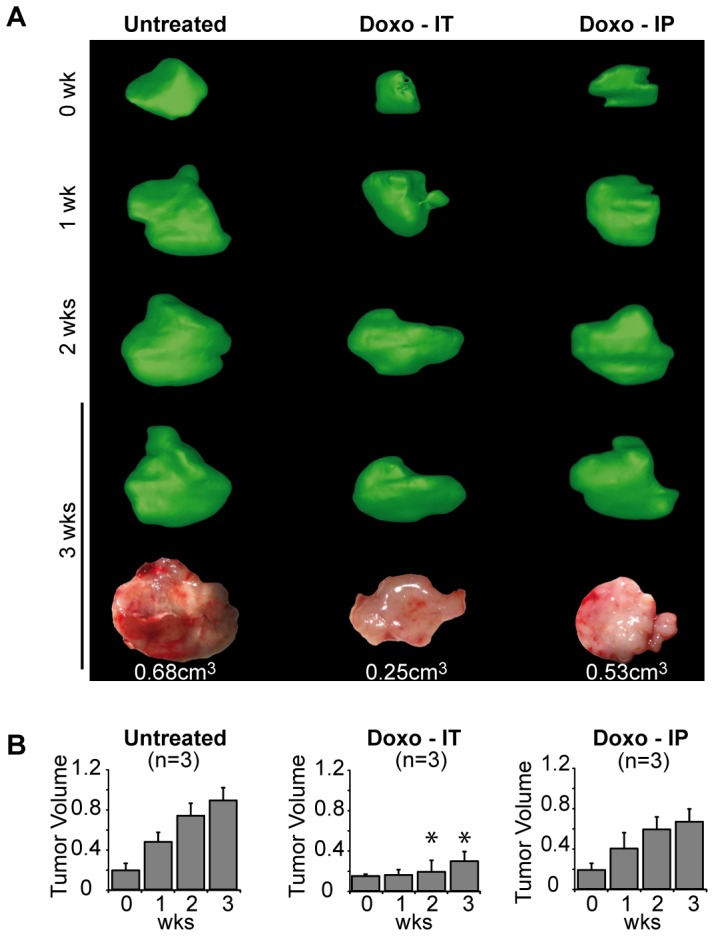
Treatment of Hep55.1C mouse HCC model by Doxorubicin. Mice bearing orthotopic HCC tumors were treated by recurrent automated robotized intratumoral injection (IT, n = 3) or manual intraperitoneal (IP, n = 3) of Doxorubicin; untreated mice (n = 3) were used as control of tumor growth. (**A**) Representative 3-weeks follow-up by µCT scan imaging and 3D reconstruction of tumor development. Volumes of the tumors resected at the end of the experiment were measured. (**B**) Tumor volume was computed at each time point from the 3D reconstructions of the tumors in untreated (left), IT (middle) or IP (right) injected mice (mean +/− SEM, star (*) indicates a difference with untreated control at the same time point with p<0.05).

Treatment started two weeks after Hep55.1C cell inoculation in a liver lobe, once tumor was detected by µCT scan. In the IT group, each of the 3 mice was subjected to robotized administration of 10 µl of Doxorubicin, at the center of the tumor. In the IP group, the same amount of drug was injected manually in the peritoneum at the vicinity of the tumor. Tumor volume was determined for each mouse at each time point by 3D reconstruction from the µCT scan data (Figure 5A). Tumors resected at the end of the experiment after 3 weeks of treatment were smaller in the IT group than in the IP and control group. µCT scan follow up demonstrated that IT injection of Doxorubicin decreased tumor growing rate by comparison to untreated mice, as tumor volume in the IT group was significantly smaller than in the control group after 2 weeks (Figure 5B). However, no statistical difference was observed after manual IP injection of Doxorubicin, tumor growth being only slightly affected by drug administration (Figure 5B). In addition, a statistical increase in apoptosis was evidenced by TUNEL assay in the tumor injected intratumorally with Doxorubicin by comparison to the non-treated control group (Figure S6).

## Discussion

Here we have developed a HCC tumor mouse model and an automated image-guided system to follow the tumor evolution overtime and evaluate the potency of anti-cancer drugs. Among all HCC animal models, tumor cell graft is most appropriate for rapid development of a single tumor nodule. One of the main limitations of the tumor graft model in immune-competent animals is tumor rejection. The Hepa1.6 cells [20] are rejected in the majority of C57BL/6J mice [29]–[36], probably due to differences between cell line and host haplotypes. On the contrary, we observed that the Hep55.1C cell line, deriving from a C57BL/6J tumor [17], is tumorigenic after subcutaneous, intra hepatic or intra splenic injection, in 100% of both male and female mice. This model, based on the Hep55.1C cells harboring a mutation in the β-catenin pathway [17], [37] represents a reproducible HCC model in the C57BL/6J mouse strain, which has a low susceptibility to spontaneous cancers.

At the histological level, these tumor cells resemble a poorly differentiated human HCC, with small sized cell, a high proliferation index, and duct like structures. A high level of fibrosis was also evidenced in the tumor as reported for other syngeneic or xenogeneic liver tumor models in mouse [38], [39]. At the molecular level, an increased expression of inflammatory and pro-fibrotic genes has been observed, in line with hepatic stellate cell activation. Recent studies demonstrated that hepatic stellate cells not only play a major role in fibrosis, but also in liver development, regeneration, and cancer, notably by promoting HCC cell growth [40]–[42]. The Hep55.1C tumors being vascularized, expressing some human cancer and HCC markers like osteopontin, cyclin D1 and AFP, the model seemed suitable to analyze the effect of anti-cancer or anti-fibrotic drugs.

Several therapies in preclinical development or evaluated in clinical trials require intratumoral administration [4]–[6], [43]–[45] that helps to reduce toxicity on normal tissue and improve treatment efficacy as concentration is locally increased. In rodents, one of the main constraints is the small size of the tumor necessitating to perform a laparotomy [35] for direct injection in orthotopic tumors. We here have designed a dedicated animal restrainer bed allowing both reproducible positioning of the animal in the µCT scan for longitudinal follow-up, and firm animal restrainment for accurate percutaneous needle insertion. Our image guided system for percutaneous drug administration did not damage surrounding tissues (see also [18], [19]).

By combining µCT imaging and 3D reconstructions [15],[21], we localized and computed the volume of liver tumors after intrahepatic or intrasplenic graft of Hep55.1C cells. As an example, we demonstrated that the robotized intratumoral injection of low amount of Doxorubicin, a drug used notably for transarterial chemoembolization of HCC [46], significantly reduced tumor growth by around 60% after 3 weeks, whereas manual intraperitoneal administration of the same amount of drug has no significant effect on tumor evolution.

The high accuracy of the system would also allow to perform recurrent tumor micro biopsy to analyze on the same animal at the molecular level, the evolution of specific markers, notably in response to drug administration. Due to the high level of fibrosis in the tumor, this model could also be used to test anti fibrotic drugs. Further developments will include the reduction of the duration of the robotized procedure for routine use on larger groups of animal, and also the development of a microbiopsy needle to retrieve biological samples from recurrent robotized biopsies.

## Materials and Methods

### Ethics statement

The project was approved by the local ethics committee (ICOMETH), under the permit number 38.2011.01.011. All experiments were performed under gaseous anesthesia, and animals were euthanized by overdosage of anesthesia followed by cervical dislocation.

### Cell lines

Hep-55.1C derived from DENA induced HCC in C57BL/6J mouse [17] was obtained from Cell Lines Service (Eppelheim, Germany) and maintained in Dulbecco's modified Eagle's medium supplemented with 4.5 g/L glucose and 10% fetal bovine serum in a 5% CO2 humidified chamber. The Hepa1.6 cell line derived from an HCC tumor in a C57L/J mouse [20] was obtained from ATCC and maintained in Dulbecco's modified Eagle's medium supplemented with 1 g/L glucose and 10% fetal bovine serum in a 5% CO2 humidified chamber.

### Cytotoxicity assay

24 h before treatment, 1.5 10^3^ Hep55.1C or Hepa1.6 cells were seeded in 96 well plates. 50–70% confluent cells were treated with Sorafenib (Nexavar, Bayer), Doxorubicin (Adriamycin, Pfizer) or DMSO for 72 h and MTT (Sigma-Aldrich) dissolved in DMEM was added to the cells for 4 h. MTT formazan crystals were then dissolved in isopropanol: HCl (10∶1) containing 0.01% NP-40. Background absorbance at 690 nm was subtracted from the measurement at 570 nm.

### Mouse Hepatoma tumor model

C57BL/6J mice (Janvier Labs, France) experiments were realized under anesthesia, following FELASA recommendations and local ethics committee approval. For subcutaneous tumor, 10^6^ cells resuspended in PBS (Sigma-Aldrich) where injected subcutaneously in the back of anaesthetized mice. For orthotopic tumor graft, 2×10^6^ Hep55.1C cells were injected in the left liver lateral lobe of anaesthetized 8 week-old mice after midline laparotomy as described in [21]. Diffuse HCC was induced after injection of 2×10^6^ Hep55.1C cells in PBS into the spleen of C57BL/6J mice following subcostal incision under gaseous anesthesia.

### Mouse Scanning procedure

MicroCT images were obtained on a micro CAT II scanner (Imtek Inc, Siemens) at 80 kVp X-ray voltage and 500 µA anode current under general gaseous anesthesia with isoflurane (Abbott). Respiratory-gated images were acquired with a voxel size of 119×119×119 µm, corresponding to a scanned volume of 6.1×6.1×6.1 cm to encompass the whole registration cover.

Four hours before imaging, animals were injected intraperitoneally with 6 µl/g Fenestra LC (ART, Canada) liver contrast agent. This polyiodinated contrast agent is taken-up specifically by normal hepatocytes via the ApoE cell surface receptors [47], [48]. Five min before µCT scan the mouse was anaesthetized, the abdomen was shaved and disinfected. The mouse was then positioned on the restrainer bed and fastened with a sterile sticky operative field. A pressure pad placed on the back of the mouse and connected to a pressure transducer served to gate µCT imaging and needle insertion on animal respiration.

µCT images exported by the AMIRA software were processed with VR-Render (available at http://www.ircad.fr/softwares) for visualization and planning or with 3DVPM for tumor segmentation, 3D reconstruction and to compute tumor volume [49], [50].

### Histology

Excised tumors and normal liver were fixed in 10% formalin and embedded in paraffin after dehydration in ethanol and xylene substitute. 5 µm thick sections were stained with Eosin and Haematoxylin for morphometric analyses. Reticulin was stained with silver according to Gordon and Sweet [51], collagen was stained with Sirius Red (Euromedex, France). For immunofluorescence, tissue was frozen in OCT embedding medium above liquid nitrogen. After permeabilization/fixation in 4% PFA, 0.1% Triton X-100 in PBS, 5 µm thick cryosection were saturated with PBS-5% BSA, and incubated with primary anti-PECAM-1 or anti-Ki-67 (Santa Cruz Biotech) and secondary AlexaFluor 488 anti-Rabbit antibodies. Lipid vesicles were stained using Oil Red O (Sigma-Aldrich, France) on frozen section. For apoptosis detection, TUNEL assay was performed on paraffin sections using the Apoptag Red in situ detection kit (Merck Millipore, France), according to the manufacturer recommendations. Images were acquired with an Axiophot microscope equipped with a CDD camera (Axiocam, Carl Zeiss) and processed with Photoshop CS4.

### RT-PCR

Tumor and normal liver samples were homogenized in a Fastprep apparatus (MP Biomedical) in Lysis Matrix B tubes (MP Biomedical) containing lysis buffer (Sigma). RNAs were purified with the mammalian Genelute kit from Sigma, according to the manufacturer recommendations. 3 µg of RNA were used as template for reverse transcription with random hexamer in the presence of 200 units of Superscript II reverse transcriptase (Invitrogen). cDNA were analyzed by real time quantitative PCR on a Chromo4 (Biorad), using Quantitect mastermix (Qiagen). Primer sequences for the PCR were as follows: 36B4: 5′-GAGGTCACTGTGCCAGCTCA-3′ and 5′-GAAGGTGTACTCAGTCTCCA-3′; AFP: 5′-GGCAAAGCCCTACAGACCA-3′ and 5′-TAAACGCCCAAAGCATCAC-3′; ALB: 5′-CCCTGTTGCTGAGACTTGCT-3′ and 5′-CTGAGGTGCTTTCTGGGTGT-3′; ASMA: 5′-GGCTGTGCTGTCCCTCTATG-3′ and 5′-TCTCACGCTCGGCAGTAGTC-3′; CCND1: 5′-AACTACCTGGACCGCTTCCT-3′ and 5′-GCTTGTTCTCATCCGCCTCT-3′; COLA1: 5′-TTTGGAGAGAGCATGACCGA-3′ and 5′-AAGTTCCGGTGTGACTCGTG-3′; IL-6: 5′-AGGATACCACTCCCAACAGAC-3′ and 5′-AGTGCATCATCGTTGTTCATAC-3′; FN1: 5′-CCACCTCGAGCCCGTTATAG-3′ and 5′-GCAGAGGCTGCAGGGTAGTA-3′; MMP3: 5′-CACGAGGAGCTAGCAGGTTA-3′ and 5′-TCCAACTGCGAAGATCCACT-3′; PDGFB: 5′-CCGGTCCAGGTGAGAAAGAT-3′ and 5′-AATAACCCTGCCCACACTCT-3′; SPP1: 5′-GCTTGGCTTATGGACTGAGG-3′ and 5′-CTCTCCTGGCTCTCTTTGGA-3′; TGFB: 5′-ATTCAGCGCTCACTGCTCTT-3′ and 5′-ACTTCCAACCCAGGTCCTTC-3′.

### Robotic System Design

The robotized procedure for image-guided percutaneous injection has been described [18]. Briefly, a specific mouse restrainer bed (Figure 1A–C) was developed and prototyped by stereolithography (Initial, France) in ABS-like epoxy resin (Protogen 18420) to maintain the animal during the different steps of the procedure. Animal respiration is monitored via a custom developed software using a pressure pad placed on the back of the animal and connected to a Unik5000 pressure transducer (GE, France) linked to a USB multifunction I/O NI 6800 (National Instruments, France). Following mouse imaging in the µCT scan, the needle insertion is performed in a robotized cell according to the trajectory defined in the µCT scan images, from the skin entry point to the center of the tumor. The robotized system is composed of a 6-degree of freedom (DOF) robotic arm RV-1A (Mitsubishi), holding a needle injector and able to reproduce the motion of a human arm with high accuracy and excellent repeatability (+/−0.02 mm). Two stereoscopic cameras (AVT Marlin, Stemmer Imaging, France) are fixed on a tripod to observe the mouse bed with an angle of about 55°. A grid projector fixed on the top of the robotized cell projects structured light on the bed as detailed in [18]. The needle injector is composed of a high speed linear motorized stage M-663 (PI France S.A.S) driven by a C-867 controller (PI France S.A.S). A disposable 29G Myjector syringe (Terumo/Thermo Fisher, France) filed with the drug is fixed on the needle injector prior to the calibration of the system.

### Non-invasive follow-up of HCC evolution and intratumoral robotized drug administration

Nine mice bearing an orthotopic Hep55.1C hepatoma tumor were randomly assigned to 3 groups. IP mice received every week 10 µl of a solution of Doxorubicin at 2 mg/ml intraperitoneally under anesthesia; robotized intratumoral injection of Doxorubicin was applied to IT mice, and untreated mice were used as control. Tumor volume was determined every week by µCT imaging and 3D reconstruction of the tumors using 3DVPM [49]. Mean tumor volumes and standard deviations were determined for each group at each time point. When tumor volume reached 5% of total body weight, as determined by µCT imaging, (assuming 1 cm^3^ of tumor weights 1 g) in one mouse, the experiments was stopped, all mice were sacrificed, and the tumors dissected.

## Supporting Information

Figure S1 **Orthotopic HCC development after inoculation of hepatoma cells in the liver or in the spleen.** Hep55.1C cells were surgically injected either in the liver left lateral lobe (**A, C**) or in the spleen (**B, D**) of C57BL/6J mice. (**A**) Macroscopic appearance of a unique tumor nodule (TN) 6 weeks after Hep55.1C cell inoculation in the liver (L). (**B**) Macroscopic appearance of multiple sub capsular tumor nodules (TN) 6 weeks after Hep55.1C cells injection in the spleen. (**C, D**) Contrast enhanced µCT imaging was performed 6 weeks after Hep55.1C cell inoculation. Hypodense tumor nodules (TN) in normal liver lobes (L) and in the spleen (S) are delineated by dotted lines (scale bar: 3 mm).
(TIF)

Figure S2 **Mice survival after intrahepatic Hep55.1C cell injection correlates with increased tumor volume determined by µCT-scan.** The evolution Hep55.1C orthotopically grafted mice (n = 20) was followed over 18 weeks (black line). Tumor volume (dashed line) was measured on a subset of animals (n = 6) by contrast enhanced µCT scan. For ethical reasons, animal were sacrificed when tumor size exceeded the maximal ethical volume or when humane endpoints were reached.
(TIF)

Figure S3 **Histological characterization of Hep55.1C tumors.** (**A**) Oil red O staining of lipid vesicles in Hep55.1c tumor 2 weeks after graft. (**B**) Proliferative tumor cells evidenced by immunofluorescent staining of the nuclear Ki-67 cell proliferation marker (green). Nuclei were counterstained with DAPI (blue). (**C**) Tumor vascularization evidenced by PECAM-1 endothelial cell marker immunofluorescent staining (green) 5 weeks after graft. (**D**) Silver staining of reticulin fibers in Hep55.1C tumors demonstrating the presence of fibrosis (black arrow) surrounding duct like structures (white arrow) 9 weeks after graft.
(TIF)

Figure S4 **Fibrosis induction by diethylnitrosamine.** Fibrosis was induced in the liver of C57BL6/J mice prior to Hep55.1C cell graft by weekly DENA injection. (**A**) Liver fibrosis evidenced by increased Sirius red staining of the collagen fibers (black arrow). (**B**) Western Blot demonstrates the increased expression of ASMA following activation of Hepatic Stellate Cells by DENA.
(TIF)

Figure S5 **In vitro evaluation of image guided robotized needle positioning.** (**A**) Volume Rendering view from µCT scan of the mouse restrainer bed filed with an 80%parafin: 20%solvent phantom. 22 virtual targets were defined in this phantom (yellow sphere, only 7 targets (T1 to T7) are represented). (**B**) Axial view of the needle track observed on a post-operative µCT scan of the phantom after robotized positioning. The distance between the targeted position (T1) and the needle tip stop was measured to calculate the precision of the procedure. (**C**) Box plot representing the positioning precision in the three dimensions for from 3 independent experiments on 22 targets (mean: white dash; 1st and 3rd percentile: white rectangle; min/max values: black dashes).
(TIF)

Figure S6 **Intratumoral injection of Doxorubicin increases apoptosis.** Apoptosis was evidenced in tumors from the non-treated control group (**A, B**) and from the intratumorally injected group (**C, D**) by indirect TUNEL assay. Nuclei were counterstained with DAPI. (**E**) Mean positive nuclei observed per microscope field of view at magnification 40x.
(TIF)
