# Supplementary figures and images for: Design and Development of a Robotized System Coupled to µCT Imaging for Intratumoral Drug Evaluation in a HCC Mouse Model
Source: PLoS One. 2014 Sep 9;9(9):e106675. doi: 10.1371/journal.pone.0106675 (PMC4159281; doi:10.1371/journal.pone.0106675)

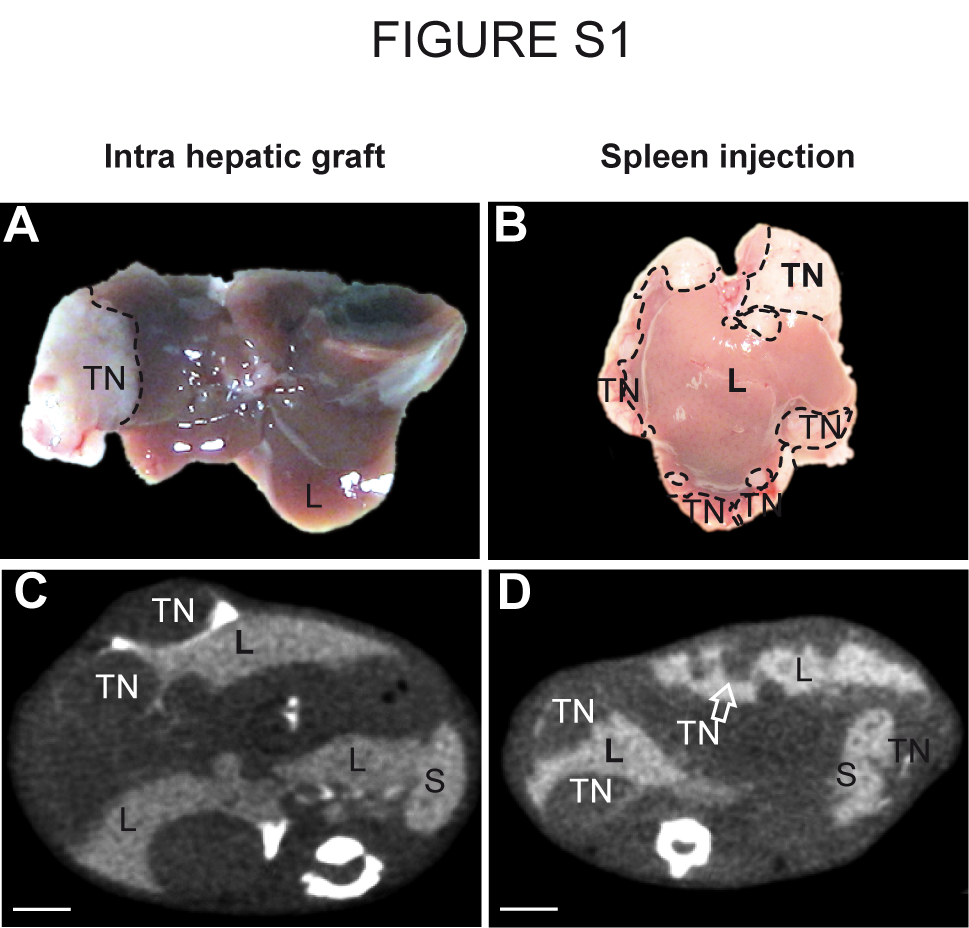

Supplement: Figure S1 — Orthotopic HCC development after inoculation of hepatoma cells in the liver or in the spleen. Hep55.1C cells were surgically injected either in the liver left lateral lobe (A, C) or in the spleen (B, D) of C57BL/6J mice. (A) Macroscopic appearance of a unique tumor nodule (TN) 6 weeks after Hep55.1C cell inoculation in the liver (L). (B) Macroscopic appearance of multiple sub capsular tumor nodules (TN) 6 weeks after Hep55.1C cells injection in the spleen. (C, D) Contrast enhanced µCT imaging was performed 6 weeks after Hep55.1C cell inoculation. Hypodense tumor nodules (TN) in normal liver lobes (L) and in the spleen (S) are delineated by dotted lines (scale bar: 3 mm). (TIF) [file pone.0106675.s001.tif]

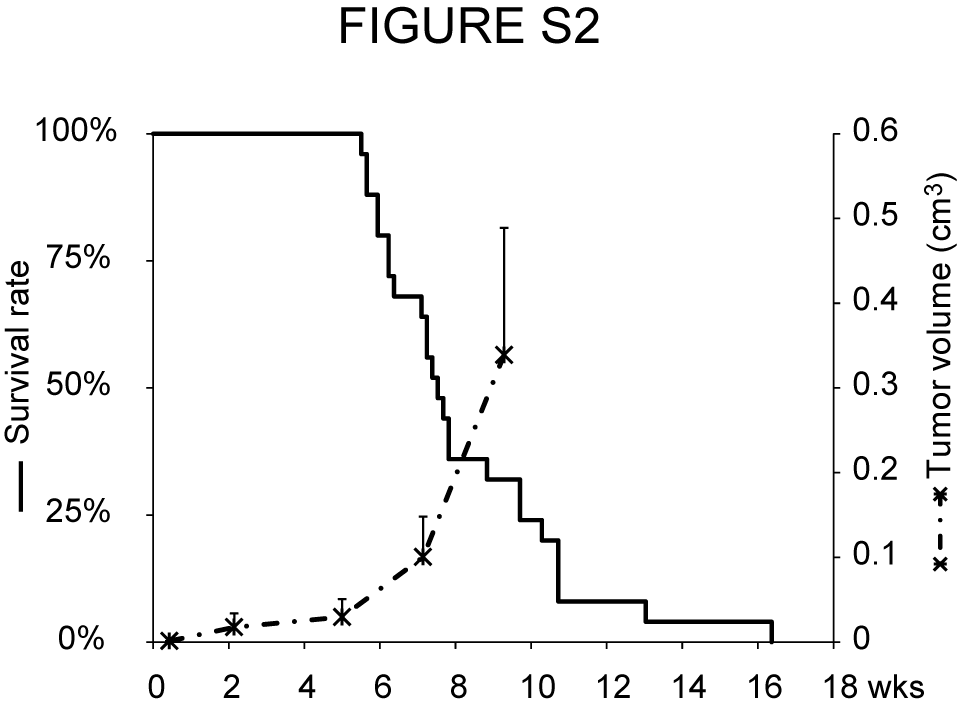

Supplement: Figure S2 — Mice survival after intrahepatic Hep55.1C cell injection correlates with increased tumor volume determined by µCT-scan. The evolution Hep55.1C orthotopically grafted mice (n = 20) was followed over 18 weeks (black line). Tumor volume (dashed line) was measured on a subset of animals (n = 6) by contrast enhanced µCT scan. For ethical reasons, animal were sacrificed when tumor size exceeded the maximal ethical volume or when humane endpoints were reached. (TIF) [file pone.0106675.s002.tif]

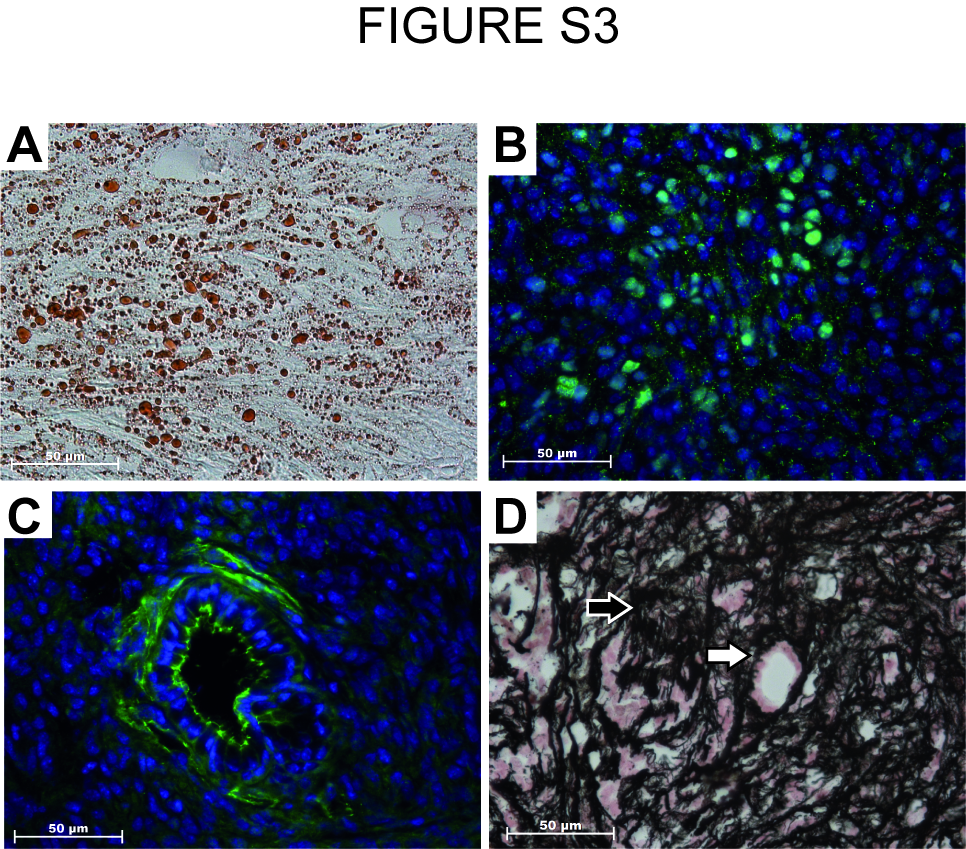

Supplement: Figure S3 — Histological characterization of Hep55.1C tumors. (A) Oil red O staining of lipid vesicles in Hep55.1c tumor 2 weeks after graft. (B) Proliferative tumor cells evidenced by immunofluorescent staining of the nuclear Ki-67 cell proliferation marker (green). Nuclei were counterstained with DAPI (blue). (C) Tumor vascularization evidenced by PECAM-1 endothelial cell marker immunofluorescent staining (green) 5 weeks after graft. (D) Silver staining of reticulin fibers in Hep55.1C tumors demonstrating the presence of fibrosis (black arrow) surrounding duct like structures (white arrow) 9 weeks after graft. (TIF) [file pone.0106675.s003.tif]

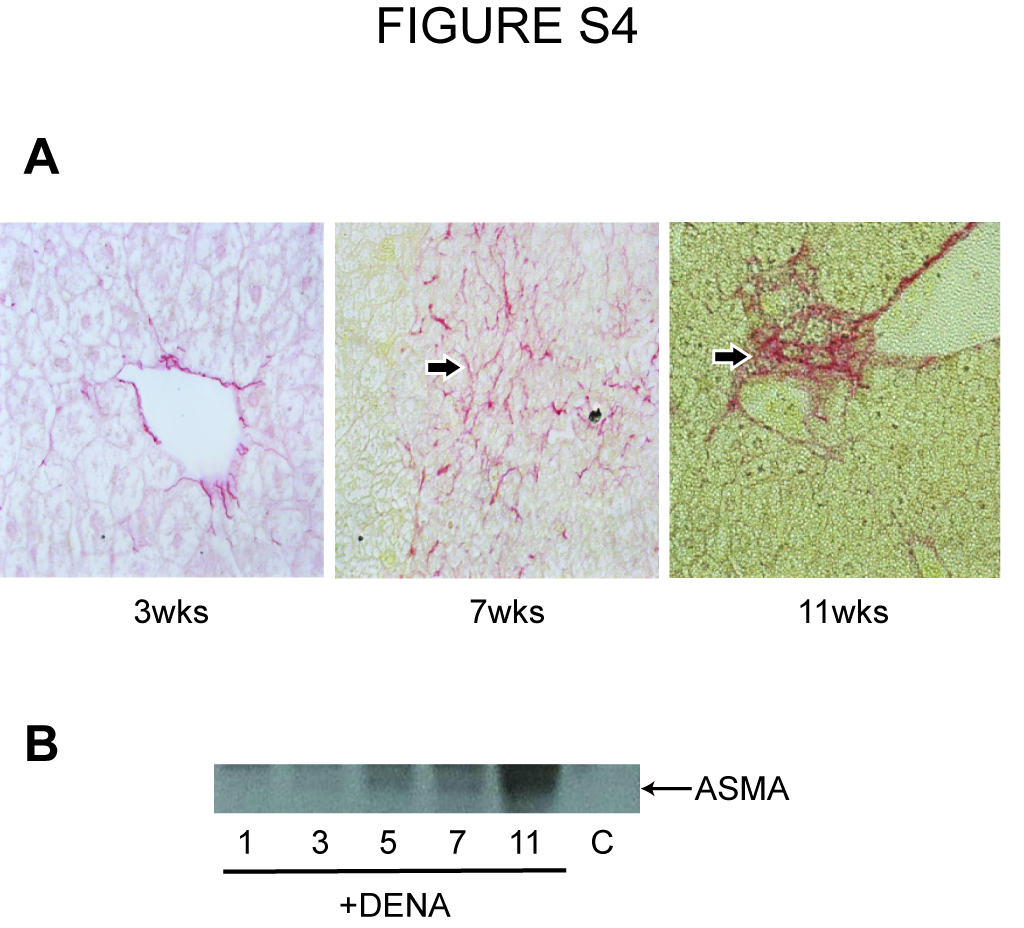

Supplement: Figure S4 — Fibrosis induction by diethylnitrosamine. Fibrosis was induced in the liver of C57BL6/J mice prior to Hep55.1C cell graft by weekly DENA injection. (A) Liver fibrosis evidenced by increased Sirius red staining of the collagen fibers (black arrow). (B) Western Blot demonstrates the increased expression of ASMA following activation of Hepatic Stellate Cells by DENA. (TIF) [file pone.0106675.s004.tif]

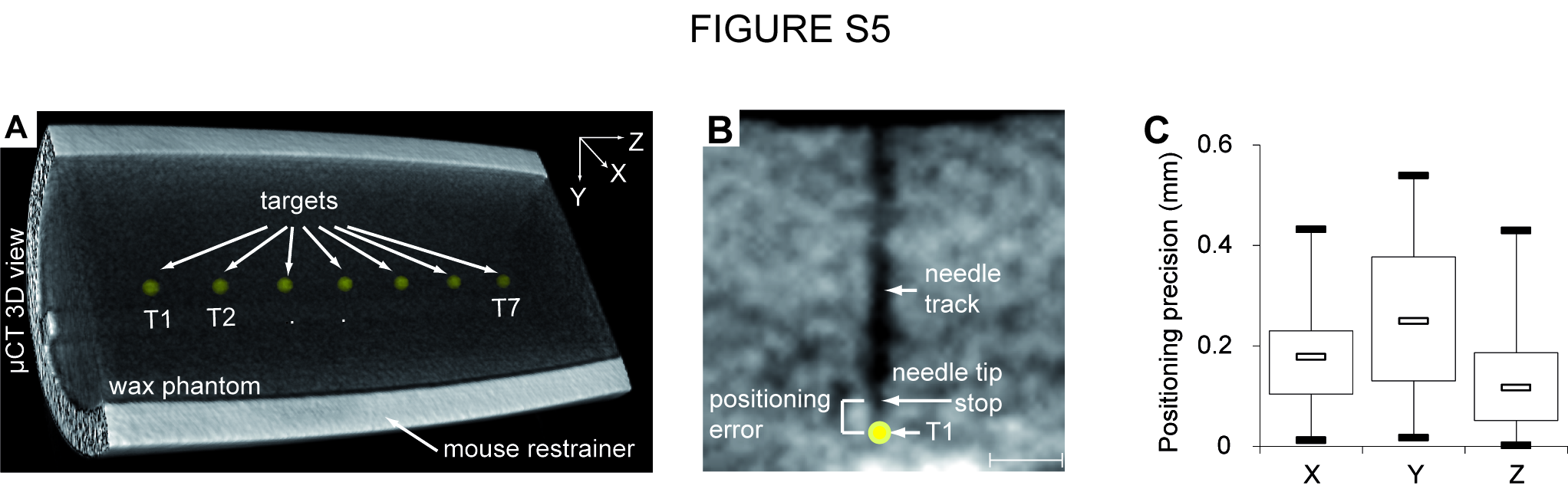

Supplement: Figure S5 — In vitro evaluation of image guided robotized needle positioning. (A) Volume Rendering view from µCT scan of the mouse restrainer bed filed with an 80%parafin: 20%solvent phantom. 22 virtual targets were defined in this phantom (yellow sphere, only 7 targets (T1 to T7) are represented). (B) Axial view of the needle track observed on a post-operative µCT scan of the phantom after robotized positioning. The distance between the targeted position (T1) and the needle tip stop was measured to calculate the precision of the procedure. (C) Box plot representing the positioning precision in the three dimensions for from 3 independent experiments on 22 targets (mean: white dash; 1st and 3rd percentile: white rectangle; min/max values: black dashes). (TIF) [file pone.0106675.s005.tif]

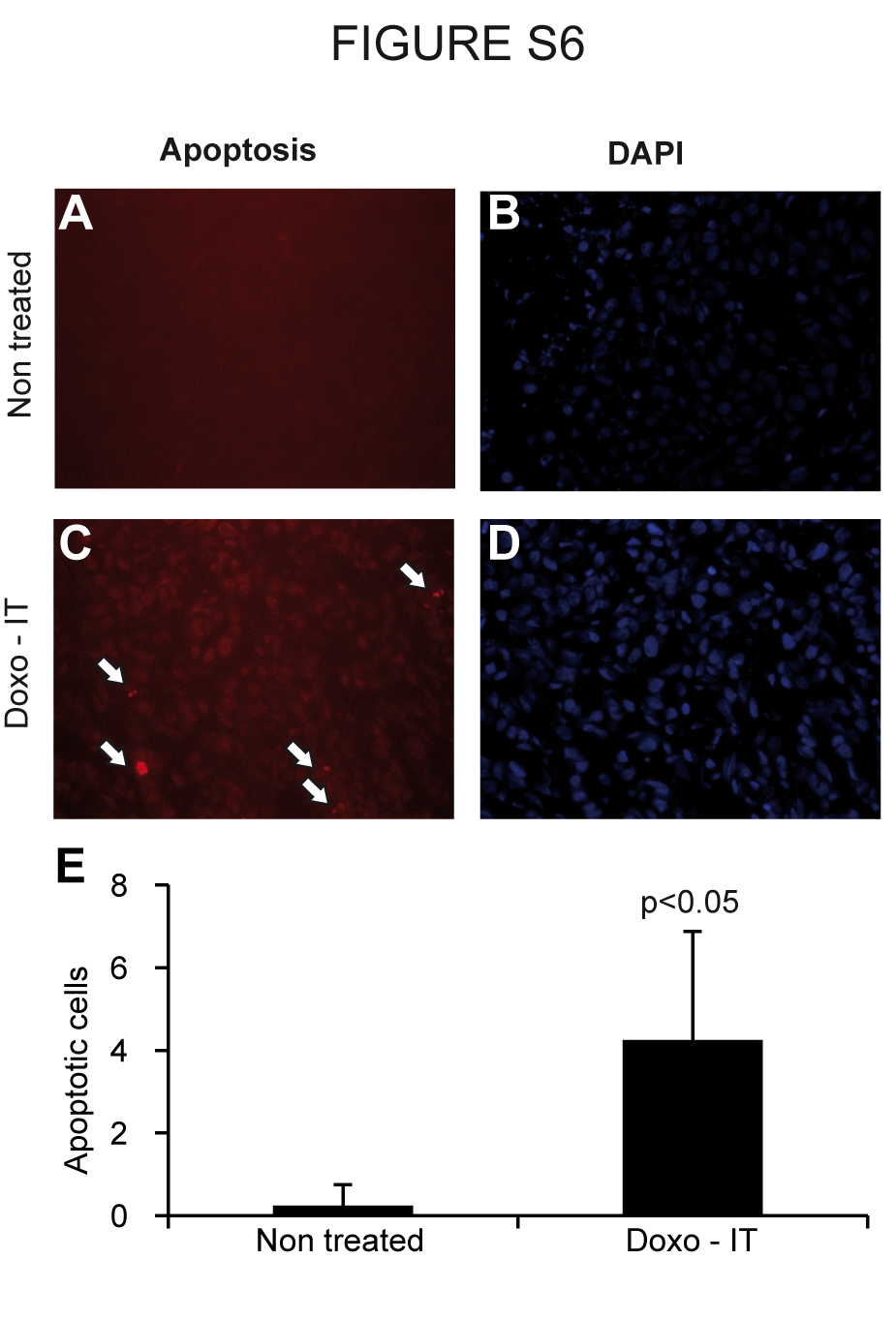

Supplement: Figure S6 — Intratumoral injection of Doxorubicin increases apoptosis. Apoptosis was evidenced in tumors from the non-treated control group (A, B) and from the intratumorally injected group (C, D) by indirect TUNEL assay. Nuclei were counterstained with DAPI. (E) Mean positive nuclei observed per microscope field of view at magnification 40x. (TIF) [file pone.0106675.s006.tif]
